# Impact of Central Event Adjudication on the PLATO Trial Results

**DOI:** 10.31083/RCM36733

**Published:** 2025-04-27

**Authors:** Victor L. Serebruany, Wendy Ziai, Hector A. Cabrera-Fuentes, Brendon Pokov, Isabella Hwang, Thomas Marciniak

**Affiliations:** ^1^Department of Neurology, Johns Hopkins University School of Medicine, Baltimore, MD 21205, USA; ^2^HeartDrug Research LLC, West Friendship, MD 21294, USA; ^3^R&D Group, Vice Presidency for Scientific Research and Innovation, Imam Abdulrahman bin Faisal University (IAU), 31441 Dammam, Saudi Arabia; ^4^UNAM-UABJO Research Center, Universidad Autónoma Benito Juárez de Oaxaca, 68120 Oaxaca, Mexico; ^5^Department of Pharmacology, University of Maryland, College Park, MD 20742, USA; ^6^Physician, retired from FDA, Alexandria, VA 22314, USA

**Keywords:** event adjudication, clinical trial, primary endpoint, bleeding, death, myocardial infarction, stroke

## Abstract

**Background:**

This study aimed to determine the impact of central adjudication of site-reported events in patients with acute coronary syndromes treated with ticagrelor or clopidogrel in addition to aspirin within the frame of indication-seeking The PLATelet Inhibition and Clinical Outcomes (PLATO) trial. Adjudication in randomized outcome-driven trials is supposed to maintain integrity by applying uniform rules for the quality assessment of clinical events. Some preliminary data suggest an imbalance between central and site diagnoses in PLATO. We gained access to the Food and Drug Administration (FDA)-issued adjudication dataset and analyzed the evidence.

**Methods:**

Death, myocardial infarction (MI), stroke/ transient ischemic attack (TIA), bleeding, arterial thrombotic events, and cardiac ischemic events underwent central adjudication. We assessed geography, timing, impact of disagreements, and primary endpoint composition.

**Results:**

Among 18,624 trial enrollees, 10,704 central adjudications occurred across 7171 patients in 43 countries. There were 938 deaths, 2751 cases of MI, 359 strokes/TIAs, 2680 cardiac events, 130 thrombotic events, and 3782 bleeding events. The match occurred for 5451 events, while mismatches favoring clopidogrel (n = 2535) or ticagrelor (n = 2706) (*p* = 0.79) were common for major (n = 1797), moderate (n = 942), or minor (n = 735) disagreements. The central decision prevailed in 2945 cases. There was a significant (HR = 0.84; 95% confidence intervals (CI): 0.75–0.95; *p* = 0.004) adjudication delay in the 2007–2008 events but finalized in 2009. Ticagrelor was significantly less favored in 2009 than in 2007–2008 (HR = 1.19; 95% CI: 1.05–1.34; *p* = 0.005). There was a remarkably consistent match for bleeding adjudication (HR = 1.02; 95% CI: 0.83–1.25; *p* = 0.859) between treatment arms. The primary endpoint in the PLATO trial exhibited highly significant disagreement favoring ticagrelor for vascular death (HR = 2.02; 95% CI: 1.1–3.64; *p* = 0.019); MI (HR = 2.31; 95% CI: 2.79–43.94; *p* = 0.034); stroke (HR = 1.37; 95% CI: 2.66–63.28; *p* = 0.036); total events (HR = 2.51; 95% CI: 1.86–3.39; *p* = 0.01).

**Conclusion:**

Central adjudication in the PLATO trial was delayed and impacted the primary endpoint by inflating the ticagrelor benefit, resulting in drug approval. The regulatory authorities should consider independent audits when unblinding is suspected in the indication-seeking clinical trials.

## 1. Introduction

Central event adjudication in randomized controlled trials is common for multicenter international outcome-driven studies. Delegating such a critical mission for the final assessment of the site-reported events should fix the variability of definitions, contribute to comprehending and resolving complicated clinical scenarios, remove “noise” and potential bias from the totality of evidence [1, 2, 3]. However, whether or not the uniform use of central adjudication is always justified and unbiased is unclear. The PLATelet Inhibition and Clinical Outcomes (PLATO) trial enrolled 18,624 patients with unstable angina or myocardial infarction (MI) treated coronary stenting or managed medically. The patients were randomized to receive ticagrelor 180 mg loading followed by 90 mg twice daily or clopidogrel 300–600 mg loading followed by 75 mg once daily, for up to one year [4]. The primary endpoint was defined as a combination of vascular death including bleeding fatalities, MI, and stroke. These events occurred in 11.7% of patients from clopidogrel arm, compared to 9.8% of ticagrelor treated patients (HR = 0.84; 95% confidence intervals (CI): 0.77–0.92; *p* < 0.001) [4]. These favorable findings of the published trial results were challenged by the Food and Drug Administration’s (FDA) Secondary Review [5] and a Review of Complete Response [6].

Lately, the alleged clinical benefit of ticagrelor has been challenged further by mortality timing, causes, and vanished fatalities [7, 8], “friendly” pool of central adjudicators and International Central Adjudication Committee (ICAC) leadership [9], and involvement of sponsor representatives in the adjudicator selection [5, 6, 10]. Moreover, ticagrelor benefit based on site-reporting was not significant in PLATO [5, 6], while the disagreement in MI counts favoring ticagrelor was concerning [11, 12]. The latest comprehensive BMJ investigation confirmed the adjudication bias in PLATO [13]. However, several critical pieces with regard to the timing of final adjudication and primary endpoint data analyses were still unclear. We gained access to the FDA-issued adjudication dataset and analyzed the evidence focusing on geography, timing of final adjudication, disagreements, and trends for drug favoritisms in crosstabulation including primary trial endpoint components.

## 2. Methods

### 2.1 Data Retrieval

We filed a legal complaint in the US Federal Court (case 1:21-CV 01572 BAH), reached a Joined Status Report Order with the FDA and Department of Justice based on the Freedom of Information Act law. The FDA issued over 800 pages of evidence, and among other documents we were provided with the entire PLATO adjudicated event listings submitted to the FDA by the ticagrelor New Drug Application (NDA) 22-433 sponsor.

### 2.2 Patients

Study participants and procedures are described in details elsewhere [4, 5, 6]. Patients were enrolled if presented with recent (no more than 24 hours) acute coronary syndrome (ACS) onset. Among major exclusions were contraindication against clopidogrel, fibrinolytics, oral anticoagulants, the bradycardia risk, or concomitant use of a strong cytochrome P-450 3A inhibitor or inducer. Overall, 18,624 patients were enrolled, about a quarter were diabetics, over 60% underwent stent implantation, 10% underwent heart surgery and 46% received prehospital clopidogrel. The follow-up duration was restricted to 1 year. However, 23% of participants stop taking the study drug before the end of follow-up most frequently due to repeated bleedings.

### 2.3 Events

Most adjudicated events such as death, MI, stroke, and bleeding have been defined and described in detail [4, 5, 6]. Briefly, each event was characterized by an adjudication code. The ICAC evaluated data of every patient designated by a local investigator as a possible event and also all patients who underwent heart surgery during the study. The ICAC determined that some events reported by Investigators did not qualify. On occasion, the ICAC identified additional unreported events and directed the sponsor or clinical research organization to query a site to register the event for official adjudication. If the local Investigator agreed, the event was registered and processed by the ICAC. Ultimately, each event was characterized by a unique adjudication code.

### 2.4 Adjudication Database

The FDA-issued database spreadsheet contains 10,704 events. Each event is marked by trial identification unique number, country, enrolling site, patient age, gender, treatment assignments, discontinuations, outcome codes, precise dates, and causes of trial entry and exit. In addition, enrollment codes, event tracking numbers, and patient participation in Holter, pharmacokinetics, and pulmonary function sub studies were also provided. Final adjudicated event results (CADJRES) were coded as 1 – death; 2 – myocardial infarction; 3 – stroke; 4 – recurrent ischemia; 5 – severe recurrent ischemia; 6 – fatal/life-threatening bleed; 7 – major bleed; 8 – minor bleed; 9 – minimal bleed; 10 – no event; 11 – transient ischemic attack; 12 – arterial thrombotic event; and 99 – withdrawal of consent. Exact classifications and subtypes of death, myocardial infarction, and stroke were also provided for each entry.

### 2.5 Disagreements

These were identified by the mismatch between event classification from the local site and central adjudication. The FDA provided full disclosure of such mismatches including event details from the site, final adjudication results, disagreement dates, resolution by reviewers or committee (if any), and disagreement severity (minor, moderate, or major), type, and details. We summarize such upgrades and downgrades in Table 1.

**Table 1.** **List of upgrades and downgrades for adjudication in PLATO**.

Adjudicated event	Upgrade	Downgrade
Cardiac ischemic	Angina to MI	No event
Arterial thrombotic	Confirmed	No event
Stroke/TIA	TIA to stroke	No event, or stroke to TIA
Bleeding*	More severe	Less severe or no event
Myocardial infarction	Confirmed	No event, or angina
Death	Nonvascular or unknown to vascular cause	Vascular to nonvascular or unknown cause

*, per original PLATO classification (minimal, minor, major, life-threatening or fatal). PLATO, the PLATelet Inhibition and Clinical Outcomes; MI, myocardial infarction; TIA, transient ischemic events.

### 2.6 Statistics

The significant differences were defined when a two-sided alpha value was less than 0.05 uncorrected for multiple comparisons. Categorical data were assessed by frequency and percentage statistics. Chi-square calculations were conducted to test for interplay between various bleeding types (per PLATO novel bleeding scale) and endpoint components (vascular death, MI, and stroke). Unadjusted odds ratios (OR) with 95% CI were calculated and interpreted for all chi-square tests. Unpaired *t*-test with Welch’s correction has been applied to establish the disagreement differences. A chi-square test was conducted to evaluate the significance of the observed shift towards ticagrelor “benefit”. The null hypothesis (H_0_) posits that the observed shift is due to random chance alone (sporadic), while the alternative hypothesis (H_1_) suggests that the observed shift is not solely attributable to random chance (non-sporadic). All analyses were performed using SPSS Version 28 (IBM Corp., Armonk, NY, USA), with the exception of the forest plot, which was constructed in GraphPad Prism Version 10.3.1 (GraphPad Software, San Diego, CA, USA).

## 3. Results

The FDA-issued database spreadsheet contains 10,704 events that occurred across 7171 patients. There were 938 deaths, 2751 MI’s, 359 strokes/TIAs, 2680 cardiac ischemia’s, 130 arterial thromboses, and 3782 bleeds.

Geography: The comparison of the FDA-reported and their matches with the site-reported events in different countries is shown in Table 2.

**Table 2.** **Overall characteristics of event adjudication in 43 countries enrolled in PLATO**.

Country	Patients enrolled	*Events reported	Match	Favors clopidogrel	Favors ticagrelor	Minor disagreement	Moderate disagreement	Major Disagreement
Argentina	410	284	165	49	70	12	31	58
		(69.3%)	(58.1%)	(17.3%)	(24.6%)	(2.9%)	(7.6%)	(14.1%)
Australia	83	70	43	15	12	4	4	15
		(84.3%)	(61.4%)	(21.4%)	(17.1%)	(4.8%)	(4.8%)	(18.1%)
Austria	143	62	25	20	17	5	2	8
		(43.4%)	(40.3%)	(32.3%)	(27.4%)	(3.5%)	(1.4%)	(5.6%)
Belgium	170	124	60	27	37	8	7	11
		(72.9%)	(48.4%)	(21.8%)	(29.8%)	(4.7%)	(4.1%)	(6.5%)
Brazil	590	476	272	103	101	21	43	62
		(80.7%)	(57.1%)	(21.6%)	(21.2%)	(3.6%)	(7.3%)	(10.5%)
Bulgaria	451	162	88	34	40	7	17	28
		(35.9%)	(54.3%)	(21.0%)	(24.7%)	(1.6%)	(3.8%)	(6.2%)
Canada	401	247	120	61	66	21	17	48
		(61.6%)	(48.6%)	(24.7%)	(26.7%)	(5.2%)	(4.2%)	(12.0%)
China	416	220	105	66	49	18	22	44
		(52.9%)	(47.7%)	(30.0%)	(22.3%)	(4.3%)	(5.3%)	(10.6%)
Czech Republic	1021	588	251	161	176	48	53	76
		(57.6%)	(42.7%)	(27.4%)	(29.9%)	(4.7%)	(5.2%)	(7.4%)
Denmark	382	329	196	73	60	18	19	62
		(86.1%)	(59.6%)	(22.2%)	(18.2%)	(4.7%)	(5.0%)	(16.2%)
Finland	154	91	44	22	25	13	8	8
		(59.1%)	(48.4%)	(24.2%)	(27.5%)	(8.4%)	(5.2%)	(5.2%)
France	422	215	93	65	57	8	21	30
		(50.9%)	(43.3%)	(30.2%)	(26.5%)	(1.9%)	(5.0%)	(7.1%)
Georgia	519	109	44	28	37	6	13	21
		(21.0%)	(40.4%)	(25.7%)	(33.9%)	(1.2%)	(2.5%)	(4.0%)
Germany	1156	665	349	156	160	59	56	103
		(57.5%)	(52.5%)	(23.5%)	(24.1%)	(5.1%)	(4.8%)	(8.9%)
Greece	90	62	21	23	18	7	9	8
		(68.9%)	(33.9%)	(37.1%)	(29.0%)	(7.8%)	(10.0%)	(8.9%)
Hong Kong	16	10	1	5	4	1	1	3
		(62.5%)	(10.0%)	(50.0%)	(40.0%)	(6.3%)	(6.3%)	(18.8%)
Hungary	1267	593	336	121	136	32	60	103
		(46.8%)	(56.7%)	(20.4%)	(22.9%)	(2.5%)	(4.7%)	(8.1%)
India	575	210	93	59	58	8	18	30
		(36.5%)	(44.3%)	(28.1%)	(27.6%)	(1.4%)	(3.1%)	(5.2%)
Indonesia	62	34	19	8	7	1	3	8
		(54.8%)	(55.9%)	(23.5%)	(20.6%)	(1.6%)	(4.8%)	(12.9%)
Israel	636	354	171	93	90	26	30	66
		(55.7%)	(48.3%)	(26.3%)	(25.4%)	(4.1%)	(4.7%)	(10.4%)
Italy	625	275	116	80	79	16	15	42
		(44.0%)	(42.2%)	(29.1%)	(28.7%)	(2.6%)	(2.4%)	(6.7%)
Malaysia	56	68	38	20	10	0	6	18
		(121.4%)	(55.9%)	(29.4%)	(14.7%)	(0.0%)	(10.7%)	(32.1%)
Mexico	137	93	44	27	22	4	10	14
		(67.9%)	(47.3%)	(29.0%)	(23.7%)	(2.9%)	(7.3%)	(10.2%)
Netherlands	913	594	269	142	183	54	46	109
		(65.1%)	(45.3%)	(23.9%)	(30.8%)	(5.9%)	(5.0%)	(11.9%)
Norway	159	121	57	31	33	13	9	18
		(76.1%)	(47.1%)	(25.6%)	(27.3%)	(8.2%)	(5.7%)	(11.3%)
Philippines	78	70	44	13	13	4	5	18
		(89.7%)	(62.9%)	(18.6%)	(18.6%)	(5.1%)	(6.4%)	(23.1%)
Poland	2666	1381	669	333	379	76	130	206
		(51.8%)	(48.4%)	(24.1%)	(27.4%)	(2.9%)	(4.9%)	(7.7%)
Portugal	152	120	62	31	27	4	10	13
		(78.9%)	(51.7%)	(25.8%)	(22.5%)	(2.6%)	(6.6%)	(8.6%)
Romania	397	163	105	30	28	6	12	31
		(41.1%)	(64.4%)	(18.4%)	(17.2%)	(1.5%)	(3.0%)	(7.8%)
Russia	678	381	200	83	98	14	41	68
		(56.2%)	(52.5%)	(21.8%)	(25.7%)	(2.1%)	(6.0%)	(10.0%)
South Korea	120	72	22	24	26	4	3	12
		(60.0%)	(30.6%)	(33.3%)	(36.1%)	(3.3%)	(2.5%)	(10.0%)
Singapore	64	30	14	9	7	2	2	8
		(46.9%)	(46.7%)	(30.0%)	(23.3%)	(3.1%)	(3.1%)	(12.5%)
Slovakia	336	184	118	25	41	14	24	30
		(54.8%)	(64.1%)	(13.6%)	(22.3%)	(4.2%)	(7.1%)	(8.9%)
South Africa	149	129	75	29	25	9	13	24
		(86.6%)	(58.1%)	(22.5%)	(19.4%)	(6.0%)	(8.7%)	(16.1%)
Spain	314	209	110	50	49	20	18	41
		(66.6%)	(52.6%)	(23.9%)	(23.4%)	(6.4%)	(5.7%)	(13.1%)
Sweden	347	272	168	55	49	36	18	47
		(78.4%)	(61.8%)	(20.2%)	(18.0%)	(10.4%)	(5.2%)	(13.5%)
Switzerland	211	134	53	46	35	13	8	22
		(63.5%)	(39.6%)	(34.3%)	(26.1%)	(6.2%)	(3.8%)	(10.4%)
Taiwan	92	85	36	16	33	3	5	6
		(92.4%)	(42.4%)	(18.8%)	(38.8%)	(3.3%)	(5.4%)	(6.5%)
Thailand	152	136	88	21	27	14	19	12
		(89.5%)	(64.7%)	(15.4%)	(19.9%)	(9.2%)	(12.5%)	(7.9%)
Turkey	51	37	15	5	17	1	5	3
		(72.5%)	(40.5%)	(13.5%)	(45.9%)	(2.0%)	(9.8%)	(5.9%)
UK	281	166	100	30	36	18	23	35
		(59.1%)	(60.2%)	(18.1%)	(21.7%)	(6.4%)	(8.2%)	(12.5%)
Ukraine	169	76	42	18	16	8	18	26
		(45.0%)	(55.3%)	(23.7%)	(21.1%)	(4.7%)	(10.7%)	(15.4%)
USA	1413	1003	510	234	259	79	68	202
		(71.0%)	(50.8%)	(23.3%)	(25.8%)	(5.6%)	(4.8%)	(14.3%)
Total	18,624	10,704	5451	2535	2706	735	942	1797

* , as reported in the FDA-issued dataset, the numbers per country do not match since the same patient may experience several events with different adjudication results and level of potential disagreements. The site-reported events do not necessarily match the adjudication results since events may be added or rejected. The geographical distribution of such frequencies and disagreements was remarkably consistent in PLATO. About half of the enrolled patients experienced events, with the matches and disagreements being consistent as well. Importantly such massive disagreements were well balanced between treatment arms. They were mostly due to the introduction of a novel PLATO bleeding classification with 4 categories and the controversial inclusion of “enzymatic” MI’s.

### 3.1 Timing of Adjudication

We conducted the analysis of events that occurred in 2007–2008 versus 2009 dependent on the time of final adjudication. The differences by arms crosstabulation are shown in Table 3.

**Table 3.** **Timing of central adjudication by arm favoring in PLATO**.

			Arm		Total
			Clopidogrel	Ticagrelor	
Year	2007–2008	Count	679	843	1522
		% within years	44.6%	55.4%	100.0%
	2009	Count	1789	1867	3656
		% within years	48.9%	51.1%	100.0%
Total		Count	2468	2710	5178
		% within years	47.7%	52.3%	100.0%

Enrollment in PLATO started in October 2006 through July 2008. The follow-up period ended in February 2009. Hence, most events occurred during 2007–2008 especially considering follow-up restriction to 365 days. The evidence in Table 3 suggests that PLATO ICAC kept a huge pool of events to be adjudicated later in 2009 after the trial closure. There was a significant (HR = 0.84; CI: 0.75–0.95; *p* = 0.004) adjudication delay of 2007–2008 site-reported events which was finalized in 2009. Critically, ticagrelor was less favored in 2009 in comparison to 2007–2008 (HR = 1.19; CI: 1.05–1.34; *p* = 0.005).

### 3.2 Bleeding

There was a remarkably consistent bleeding event rate over time (HR = 1.02; CI = 0.83–1.25; *p* = 0.859) between treatment arms (Table 4).

**Table 4.** **Timing of bleeding adjudication in PLATO**.

			Favor		Total
			Tica	Clop	
Group year	2007–2008	Count	373	337	710
		% within group year	52.5%	47.5%	100.0%
	2009	Count	390	359	749
		% within group year	52.1%	47.9%	100.0%
Total		Count	763	696	1459
		% within group year	52.3%	47.7%	100.0%

### 3.3 Death

The disagreements between centrally adjudicated and site reported deaths over time of enrollment in PLATO are shown in Table 5.

**Table 5.** **Arm favoring and timing of death adjudication in PLATO**.

			Favor		Total
			Ticagrelor	Clopidogrel	
Group year	2007–2008	Count	99	26	125
		% within group year	79.2%	20.8%	100.0%
	2009	Count	62	33	95
		% within group year	65.3%	34.7%	100.0%
Total		Count	161	59	220
		% within group year	73.2%	26.8%	100.0%

### 3.4 Myocardial Infarction

There was no difference (HR = 0.85; 95% CI = 0.46–1.58; *p* = 0.628) in favoring in terms of MI between 2007–2008 versus 2009. However, overall, there was a consistent significant (*p* = 0.02) less MI adjudication in the ticagrelor arm (Table 6). There was a significant difference in favoring ticagrelor over clopidogrel in terms of mismatched death (HR = 2.02; 95% CI = 1.1–3.64; *p* = 0.0208). There was a significantly (*p* = 0.001) higher rate of centrally adjudicated deaths in 2009 than in 2007–2008.

**Table 6.** **Timing of MI central adjudication and favor crosstabulation in PLATO**.

			Favor		Total
			Ticagrelor	Clopidogrel	
Group year	2007–2008	Count	32	28	60
		% within group year	53.3%	46.7%	100.0%
	2009	Count	68	51	119
		% within group year	57.1%	42.9%	100.0%
Total		Count	100	79	179
		% within group year	55.9%	44.1%	100.0%

### 3.5 Disagreements

The severity of adjudication disagreements for 3 components of the trial primary endpoint is presented in Table 7.

**Table 7.** **Disagreements between central adjudication and sites for vascular death, MI and stroke in PLATO**.

			CEA result			Total
			Death-vascular	MI	Stroke	
Disagreement severity	Minor	Count	21	29	34	84
		% within Adj result	9.4%	4.4%	69.4%	9.0%
	Moderate	Count	138	425	0	563
		% within Adj result	61.9%	64.0%	0.0%	60.1%
	Major	Count	64	210	15	289
		% within Adj result	28.7%	31.6%	30.6%	30.9%
Total		Count	223	664	49	936
		% within Adj result	100.0%	100.0%	100.0%	100.0%

CEA, clinical endpoint adjudication; Adj, adjudication.

In contrast to stroke when most disagreements were minor (69.4%) the severity of mismatched for vascular death and MI was either moderate or major. In fact, the average of major disagreements hovered around 30% for all adjudicated results.

### 3.6 Primary Endpoint

There were 864 events in the ticagrelor arm and 1014 events in the clopidogrel arm constituting PLATO’s main efficacy result. Table 8 indicates favors of central adjudication exclusively for the events constituting the trial primary endpoint.

**Table 8.** **Impact of central adjudication favoring over site-reported events on PLATO primary endpoint components**.

			Endpoint			Total
			Death-vascular	MI	Stroke	
Group assignment	Ticagrelor	Count	59	73	7	139
		% within adjudication	72.0%	61.9%	63.6%	65.9%
	Clopidogrel	Count	23	45	4	72
		% within adjudication	28.0%	38.1%	36.4%	34.1%
Total		Count	82	118	11	211
		% within adjudication	100.0%	100.0%	100.0%	100.0%

**Fig. 1.**
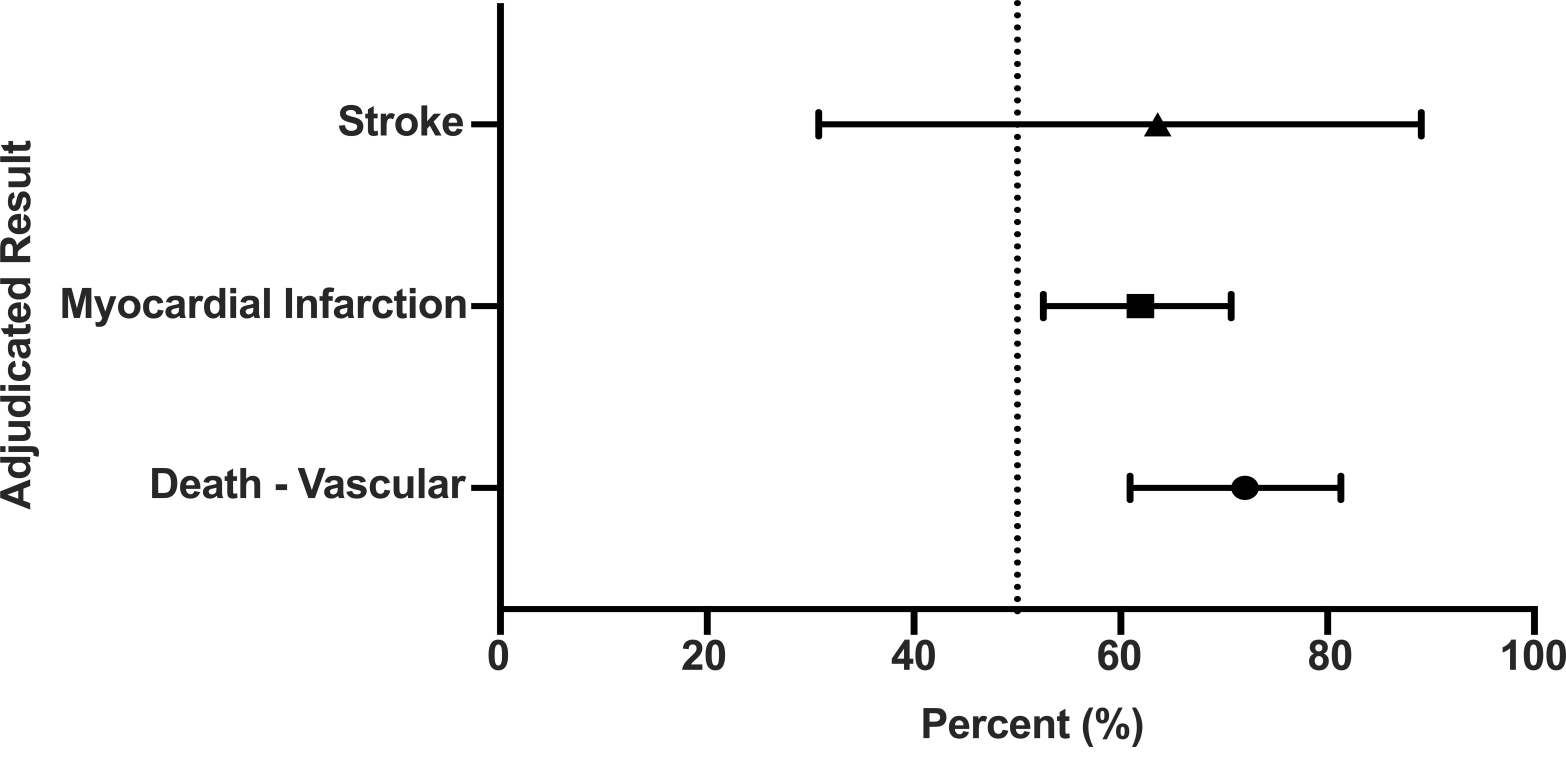
**Adjudicated primary end point results favor Ticagrelor in the PLATO study**.

With regard to favoring, there was a consistent shift towards ticagrelor advantage for all 3 primary endpoint trial components. There was relatively equal dispersion of ticagrelor “favoring” for vascular death (72.0%), MI (61.9%), and stroke (63.6%). Overall, the central decisions differed from sites in only 211 out of 1878 primary endpoint events (11.2%). However, an extra 67 endpoint events (36 vascular deaths, 28 MI’s, and 3 strokes) were adjudicated in favor of ticagrelor. Hazard Ratios for the Primary Endpoint Adjudicated Results favoring Ticagrelor in PLATO are presented in Fig. 1.

With regard to the exclusive primary endpoint events there was highly significant disagreement favoring ticagrelor for vascular death (HR = 2.02; 95% CI: 1.1–3.64; *p* = 0.019); MI (HR = 2.31 95% CI: 2.79–43.94; *p* = 0.034); stroke (HR = 1.37; 95% CI: 2.66–63.28; *p* = 0.036; and total events (HR = 2.51, 95% CI: 1.86–3.39; *p* = 0.01). Considering that 211/1878 Primary events were found to have disagreements, an extra 67/211 (31.8%) endpoint events were adjudicated in favor of ticagrelor. Given that there were 67 extra events this will happen by chance in 1 of approximately 485 trillion trials.

## 4. Discussion

The clinical validity of central event adjudication has often been a challenge since adjudicated data usually match well with the local sites, but are substantially costly [1, 2, 3, 14, 15] and adding extra bureaucratic organizational burden [2, 15]. Historically, the balance of adjudication results and severity of disagreements were kept secretive and submitted only to the regulatory authorities, especially for the landmark indication-seeking trials. The current analyses became possible because we gained access to the full adjudication database after being awarded of the Joined Status Report by the US Federal Court Order agreement with the FDA and Department of Justice.

The main finding of this report suggests that central adjudication played a pivotal role in escalating ticagrelor benefits far beyond previously described misreported deaths [7, 8]. Without ICAC activities the benefit will remain non-significant [5, 6] making regulatory approval of ticagrelor impossible. In fairness, the ICAC in PLATO was extremely busy adjudicating over 10,000 events in a short-duration megatrial. Such massive tasks were substantiated by two main reasons: the introduction of the unique trial bleeding classification and questionable inclusion of enzymatic or “triggered” MIs. The exact definitions of these two categories were somewhat unclear to the local enrolling sites but subjected to the mandatory central adjudication accounted for about half of all adjudications and disagreements in PLATO. Also, most events were adjudicated fairly including bleedings, arterial thrombosis. and cardiac ischemic events. To make it crystal clear, most deaths, MIs and strokes were also adjudicated properly, but not all of them [7, 13]. For 1878 events constituting the primary endpoint the odds of favoring ticagrelor were 2.51 times higher (95% CI: 1.86–3.39) versus clopidogrel. Importantly, all 3 components of the primary endpoint were adjudicated favoring ticagrelor potentially challenging the treatment arm blindness of ICAC leadership. Importantly, issues with unfair adjudication rendered the PLATO results do not explain the rate of death from any cause which were reported in PLATO being lower after ticagrelor (4.5% vs. 5.9% with clopidogrel; *p* < 0.001). Until the detailed exploration of the primary database entries and their corrections are being conducted, there are certain reservations about the clinical validity of ticagrelor approval. Another shortcoming of the trial central adjudication was a massive unaccounted delay when most events that occurred in 2008 were kept unadjudicated till the very end of the trial in 2009. The PLATO ICAC reserved a huge pool of events to be adjudicated later in 2009, and this delay was significant (HR = 0.84; 95% CI: 0.75–0.95; *p* = 0.004). Importantly, ticagrelor was significantly less favored in 2009 in comparison to 2007–2008 (HR = 1.19; 95% CI: 1.05–1.34; *p* = 0.005). These data corresponds well with the earlier FDA concern that there was a lack of early benefit for ticagrelor in the largest post-stenting PLATO cohort [5, 6], while the immediate spread of Kaplan-Meier (K-M) efficacy curves was so critical to achieve. However, the exact explanation of such adjudication delay is still lacking pending further investigation.

The geographical distribution of the central adjudication patterns was remarkably consistent in PLATO. About half of the enrolled patients experienced an event, with the matches and disagreements among 43 enrolling countries being similar as well. Importantly, such massive double-digit disagreements were well-balanced between treatment arms. They were mostly due to the introduction of novel PLATO bleeding scale classifying hemorrhages into 4 categories and the controversial inclusion of “enzymatic” or “triggered” MIs into the equation. It seems most investigators were not well aware of such novelties causing massive disagreements easier to comprehend. In contrast to stroke when most disagreements were minor the severity of mismatched decisions for vascular death and MI was either moderate or major. In fact, the average of major disagreements hovered around 30% for all adjudicated results. There was a heavy selective bias in PLATO ICAC constitution and governing. Importantly, ICAC for PLATO was primarily pre-planned and was not created per the FDA suggestion [5, 6, 9]. In fact, sponsor was definitely informed and potentially involved in ICAC members/reviewers selection although the details on how such communications occurred was hard to comprehend, and the ICAC regulations charter was also ‘silent on the matter’ [6]. Importantly, the FDA inspector did not find any records that explain how the ICAC selections occurred. In short, the FDA’ confirmed that the ICAC constitution was approved by the PLATO study sponsor [5, 6] what is obviously concerning. It is unclear whether any specific procedures were in place during the PLATO trial to ensure consistency and fairness in central event adjudication. That particular issue was also the FDA reviewers concern since the sponsor refused to provide any communication records among ICAC leadership, members and sponsor. In fairness, despite the friendly pool of adjudicators, we have no proof that they were unblinded or biased. Also surprising that despite outcome disparities in the United States, Russia, Georgia, Israel, and part of Ukraine (harm with ticagrelor when Clinical Research Organization (CRO) monitoring) and the other 39 countries (extreme benefit with ticagrelor when sponsor monitoring), the FDA did not institute its own independent ICAC [6], which would be reasonable considering inversed outcomes within the same trial. What matters the most is the fact that, central adjudication changed the PLATO primary endpoint results. Without the ICAC activities, the combined primary endpoint benefit of ticagrelor versus clopidogrel in PLATO was not significant (HR = 0.92; *p* = 0.095) even by applying liberal *log-rank* statistics [5, 6]. Importantly, these findings obviously may be extrapolated or confined to the specific context of the PLATO trial and may not be generalizable to other clinical trials or patient populations.

### Strengths and Limitations

There are a few strengths worth mentioning: This analysis was conducted within the framework of a governmental database that entailed mandatory event reporting. Independent specialists with an expertise on outcome data mining and advanced statistics were used to avoid any potential bias. The sample size for all events represents one of the largest single trial uniform datasets containing 10,704 site-reported entries, allowing us to make reasonable assessments of central adjudication patterns. In fact, we analyzed here a real “terra incognita” of clinical trials historically keeping adjudications and disagreements away from the public. Finally, the comprehensive in-dept independent review of primary evidence [13, 16] confirmed our concerns. There are also several limitations to this study. While various statistical methods were employed, the study did not use multivariate analysis to control for potential confounding factors, which may limit the precision and accuracy of the inferences drawn about the adjudication outcomes. As with any mega indication-seeking trial, the evidence did not contain any potential individual confounding variables making it impossible to analyze further. Applying a multivariable model that could control for baseline and follow-up variables would result in more precise and accurate inferences impacting how each individual event was adjudicated. Such analyses would have been conducted and reported if confounders and characteristics were available in the PLATO dataset. However, the FDA redacted the adjudication database, making it impossible to explore further. We also did not have any access to the ICAC communications, or most local hospital records. Finally, we have no definite proof that ICAC leadership had been unblinded by providing them with the randomization codes before the trial ended. Therefore, the heavy shift towards ticagrelor advantages may represent a play-of-chance. On the other hand, the ICAC could have been provided with biased data, e.g., incomplete for ticagrelor events but thorough for clopidogrel events. The investigation of patient-level factors that may affect outcomes is limited by the study’s lack of comprehensive individual patient data, such as baseline characteristics and treatment responses. However, the FDA provided us with the heavily redacted adjudication dataset making this task impossible to implement. In fairness, we have no definite proof that the central adjudication panel knew the randomization codes. There is a possibility that ICAC members or leadership somehow assume or guess the treatment assignment. However, the sponsor was aware of the adjudicator’s selection, and many of them had definite conflicts of interest [9]. Regardless, the consistent shift in the primary endpoint components suggests that the biases against clopidogrel were not random. This is especially true since the probability of the observed distribution may happen by chance in 1 of 485 trillion trials but is still possible.

## 5. Conclusion

Central event adjudication in PLATO was unaccountably delayed while the primary endpoint shifted towards inflated ticagrelor benefit resulting in drug approval. The regulatory authorities should consider independent audits when there is a major disagreement between event assessments changing the results of the indication-seeking clinical trials.

## Availability of Data and Materials

The datasets used and/or analyzed during the current study are available from the corresponding author on reasonable request.
